# Higher Expression of HPV16 Derived E7_LI Transcript Observed in Men With HIV and Recurrent Anal Cancer

**DOI:** 10.1002/jmv.70371

**Published:** 2025-05-03

**Authors:** Kevin J. Maroney, Yuanfan Ye, Staci L. Sudenga, Sameer Al Diffalha, Nilam Sanjib Banerjee, Sadeep Shrestha, Anju Bansal

**Affiliations:** ^1^ Department of Medicine, Division of Infectious Diseases, Heersink School of Medicine University of Alabama at Birmingham Birmingham Alabama USA; ^2^ Ob/gyn‐Maternal and Fetal Medicine, Heersink School of Medicine University of Alabama at Birmingham Birmingham Alabama USA; ^3^ Division of Epidemiology Vanderbilt University Medical Center Nashville Tennessee USA; ^4^ Anatomic Pathology, Heersink School of Medicine University of Alabama at Birmingham Birmingham Alabama USA; ^5^ Department of Biochemistry and Molecular Genetics University of Alabama at Birmingham Birmingham Alabama USA; ^6^ Department of Epidemiology, School of Public Health University of Alabama at Birmingham Birmingham Alabama USA

**Keywords:** anal cancer, cancer recurrence, HIV, HPV, RNA‐seq, transcriptome profile, treatment response, virome, virtus2

## Abstract

Squamous cell carcinoma of the anus (SCCA) or anal cancer (AC) is an understudied cancer with a high occurrence rate in people with HIV (PWH), especially men having sex with men (MSM). Furthermore, AC recurs in approximately one‐fourth of patients who undergo standard care with chemoradiation therapy (CRT). Using bulk RNA sequencing data of AC obtained from 12 patients with non‐recurrent (NR, *N* = 9) or recurrent (R, *N* = 3) cancer, we previously showed upregulated expression of key immune genes in the NR compared to the R group. Although the main causative agent of AC is high‐risk human papillomavirus (HPV), association of host and viral RNA transcript expression contributing to AC recurrence has not been extensively studied. The objective of the current study was to determine whether enrichment of specific HPV genotypes and/or HPV gene expression patterns differentiate the two groups and if any specific viral (HPV) and host (human) immune mediators correlate with each other. Using bulk RNA sequencing data and VIRTUS 2, we detected viral RNA reads mapping to seven high‐risk and six low‐risk HPV types, of which the high‐risk HPV16 observed in 83% (10/12) AC tumors (7/9 NR and 3/3 R). Rate of all HPV genomes trended toward a decrease in NR AC isolates and correlation between HPV types was more commonly observed in low‐risk ones. Analysis of HPV 16 gene expression profile showed a significantly lower positivity rate for a polycistronic transcript encoding for E7^L1 in the NR group (1/9, NR vs. 3/3, R, *p* < 0.05). An unbiased correlation analysis of HPV‐human transcript expression showed a direct correlation between HPV transcripts and human genes involved in cell growth. The data also identified human transcripts showing an inverse correlation with HPV gene expression. These included genes involved in negative regulation of growth, proliferation, and immune response. Taken together, these data indicate that concurrent analyses of viral and host factors in the same tumor can identify potential new therapeutic targets to ameliorate cancer recurrence post‐treatment.

## 1 Introduction

Due to antiretroviral treatment (ART), although people with HIV (PWH) are living longer, they also have an increased risk of developing multi‐comorbidities including cancer as chronic immune activation and inflammation persist in PWH despite suppressive ART [1].

Squamous cell carcinoma of the anus (SCCA) or anal cancer (AC) is a relatively rare cancer in the general population [2]. However, it has a significantly higher occurrence rate in PWH, especially among men who have sex with men (MSM) [2, 3] and is one of the most common non‐AIDS defining malignancies among PWH in the United States [4]. Although chemoradiation therapy (CRT) is the standard of care for AC, the 5‐year survival rate is 76%, and recurrence of the cancer in the same anatomical site, within 5 years, can be seen in ~36% of CRT‐treated patients [5]. Human papilloma virus (HPV) is the leading causative agent of AC, with approximately 90% of all cases occurring in those with detectable HPV, and with HPV16 being the most prevalent type associated with this cancer, according to the National Cancer Institute (NCI) [6].

Human papillomavirus (HPV) is a small non‐enveloped DNA virus, and its infection includes two general outcomes: (a) rapid completion of its productive life cycle, manifested as painful but benign papillomas, or (b) an unproductive phase as a subclinical infection for many years. In most patients, the infected cells, containing the unproductive viral DNA, are eventually cleared by the host immune system. But in a subset of patients infected with high‐risk HPV (such as HPV16), especially under immunocompromised conditions, expression of viral oncogenes (E6 and E7), induce dysplasia which often develop into cancers [7].

Specifically, in terms of life cycle, HPVs naturally infect actively dividing or transit amplifying (TA) cells of the basal or parabasal layers of the squamous or columnar epithelium through endocytic vesicle internalization, and eventual passage into the nucleus [8, 9, 10, 11]. The transcription of viral genes, their translation, and replication of viral DNA are tightly regulated by epithelial differentiation of the infected cells [12, 13, 14]. HPV encodes E6 and E7 proteins which bind to and inactivate or degrade master regulators of the cell cycle, p53 and pRb pocket proteins, which induce S‐phase protein expression and initiate host DNA replication in postmitotic host cells [15]. E7 also induces a prolonged G2 phase [16], which facilitates rapid multiplication of HPV DNA assisted by viral E1 and E2 proteins, in the upper differentiated strata of the epithelium [17, 18], expression of capsid proteins L1 and L2, and progeny virus packaging in the nuclei of cornified cells. Eventual liberation of the virus particles from the exfoliated infected cells initiates a new round of the viral lifecycle. However, in some individuals, under immunocompromised conditions, a part of the viral DNA of high‐risk HPV types, containing E6 and E7 coding sequences but interrupted in the E1 region, integrates into host DNA as tandem copies or singly which abolishes virus lifecycle and induces neoplastic progression [19, 20, 21, 22, 23, 24, 25, 26, 27, 28, 29, 30].

Our prior work using bulk RNA sequencing data of AC obtained from 12 patients with either non‐recurrent (NR, *N* = 9) or recurrent (R, *N* = 3) cancer showed upregulation of key immune gene expression‐related signatures in the NR compared to the R group [31]. It is well known that about 90% of AC is caused by high‐risk HPV, in most cases HPV16, 18 or occasionally HPV33 [32], but the role of other HPV types in AC occurrence and recurrence is unknown, specifically among PWH; thus, evaluating this could be important for diagnostic and/or prognostic AC biomarker discovery. The clinical detection of HPV commonly uses hybrid capture or PCR‐based assays [33, 34, 35]. However, determining viral genotypes and their gene expression patterns from bulk RNA sequencing has not been feasible until recently due to paucity of appropriate computational tools as viral reads typically comprise less than 0.01% of total mRNA in any given human sample. Such analyses are now feasible with the newer next‐generation sequencing‐based methods which provide high sequencing depth and availability of viral analysis enabling computational tools such as Viral Transcript Usage Sensor version2 (VIRTUS 2) as used in this study [36]. In summary, although previous work on many HPV‐associated cancers including AC have examined the host gene expression or HPV types by using bulk RNA sequencing and PCR based assays, respectively, prior studies do not typically examine in tandem (a) both the host transcriptome and HPV virome of AC in the same sample by using the same bulk RNA sequencing data; and (b) determine associations between specific host and viral genes.

In the current study, we used VIRTUS2 to determine the HPV virome in AC samples with an overall study objective to determine whether enrichment of specific HPV genotypes and/or HPV gene expression patterns differentiate the R and NR groups to identify potentially novel biomarker(s) of AC recurrence. Additionally, we sought to characterize the correlation landscape of interactions between the HPV16 genotype and human transcript gene expression patterns to identify gene signatures associated with AC treatment outcome.

## 2 Results

Formalin‐fixed paraffin‐embedded (FFPE) tissues from those with NR and R AC were utilized for this study and subjected to paired‐end bulk RNA‐sequencing, as previously described [31]. VIRTUS2 was used to map these reads to a human reference first before mapping those reads (which did not match any human source) to a reference containing the complete genome sequences of all HPV genotypes to identify the different HPV types present in these samples. We also examined if we could detect specific spliced transcripts of HPV16 that differed between the R and NR groups. A graphical overview of the viral analysis approach used is shown in Supporting Information: Figure 1.

### 2.1 Higher Number of HPV Genotypes (Both Low and High‐Risk) Are Observed in NR AC Isolates

**Figure 1:**
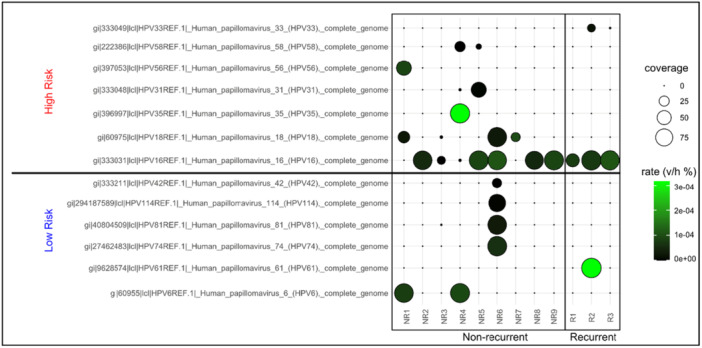
HPV16 is the predominant HPV type observed in anal cancer isolates. Unmapped nonhuman reads were mapped to all HPV types included in concatenated Virtus2 reference genomes through a modified version of the Virtus2 paired‐end workflow using a wrapper to define sample groups. Coverage and viral to human rate (v/h mapped read %) were determined for all HPV types and those which were detected are shown as indicated. The full name and source of each reference sequence are provided. Significance *p* value results of Mann−Whitney's *U*‐test performed internally through Virtus2 between indicated groups is also shown on left side of graph. HPV types identified are also divided into high‐risk and low‐risk.

All HPV complete genome references were originally sourced and are publicly available through the Papillomavirus Episteme (PaVE) or NCBI (Supporting Information: Table 1). The rate of viral reads to human reads was low even at the highest rate in these samples (max rate v/h [rate viral/human] reads of 3 × 10^−4^). Despite this, in our cohort, most samples demonstrated coverage of viral reads mapping to high‐risk HPV16 in addition to a diverse range of other high and low‐risk HPV types (Figure 1). Due to the small sample size, there was no single HPV type which was found to change significantly between the two groups, however, as expected, HPV16 was observed in most samples (10/12, 83%).

**Figure 2:**
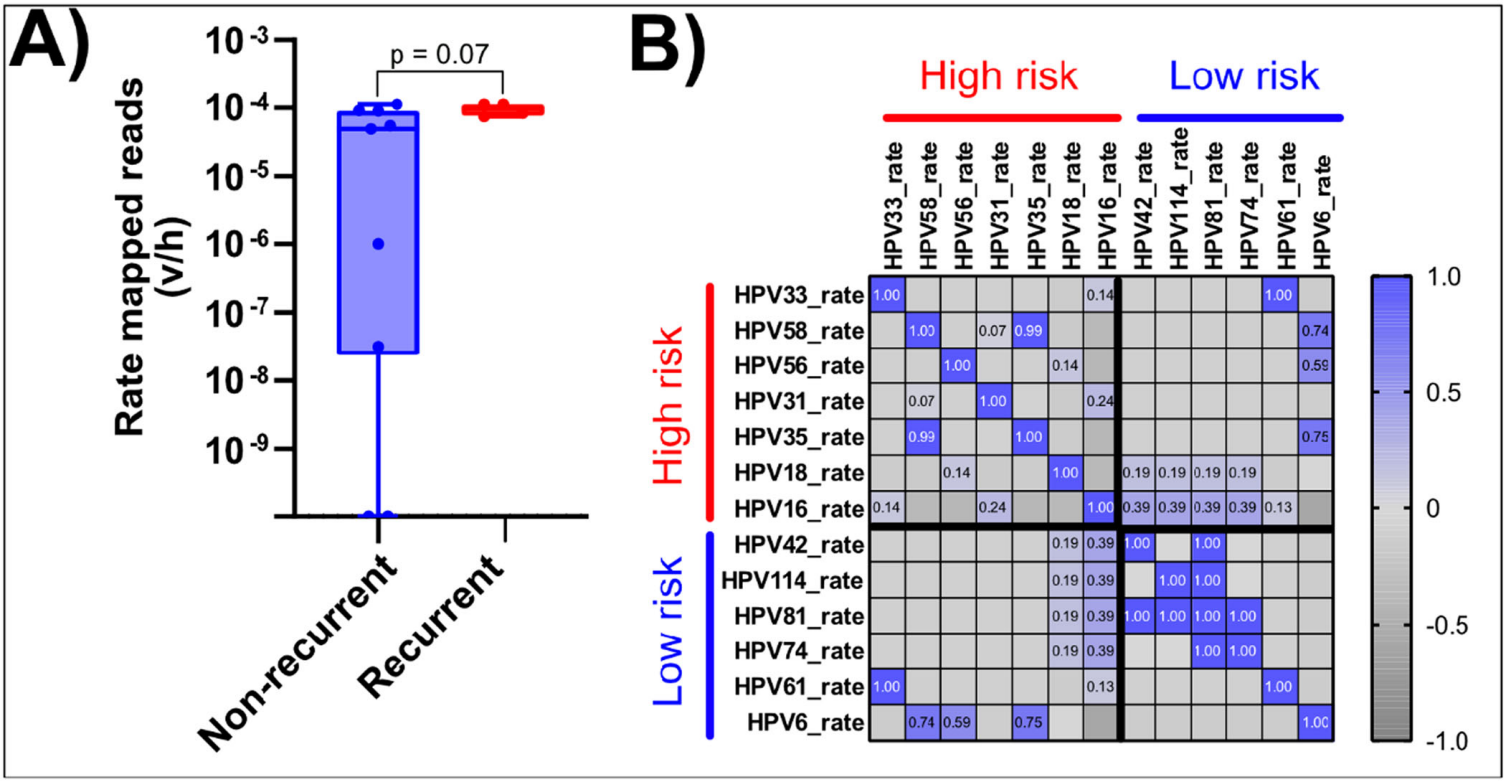
Rate of all HPV genomes trends toward increase in recurrent anal cancer isolates and correlation is more commonly observed in low‐risk HPV types. (A) Box‐plot graph representing the rate of HPV16 in both groups, *p‐*value represents the Mann−Whitney *t*‐test comparison between groups. (B) Correlation matrix representing the correlation between the presence of each of the two compared HPV types (high and low risk). Numbers indicated within each box represent the *r* value for correlation. Higher numbers and deeper blue indicate a more positive correlation. HPV types are separated into high or low risk.

While not statistically significant, the number of HPV types observed in NR isolates appeared to be higher (median # positive HPV types 3 in NR, 2 in R) than in R isolates (Supporting Information: Figure 2A) and average rate mapped reads (v/h) for all samples appeared to be higher in R isolates (Supporting Information S1: Figure 2B). The latter increase was driven most notably by HPV16, which increased in samples from the NR group (median average rate was 5.52 × 10^−5^) to those which eventually progressed to R AC (median average rate 8.26 × 10^−5^; Figure 2A, *p* = 0.07). Lastly, we determined whether the presence of certain HPV types correlates with the presence of others (Figure 2B). This correlation was more commonly observed in low‐risk types (HPV 81 and 42, 81 and 114, 81 and 74), while in the high‐risk group, this was only seen for HPV 35 and 58. It should also be noted that each of the non‐HPV16 and ‐HPV18 types (the high‐risk types most commonly observed in carcinomas AC) were only observed in a single sample.

### 2.2 HPV16 E7‐L1 Polycistronic Transcript Is Significantly Enriched in R Cancer Isolates

**Figure 3:**
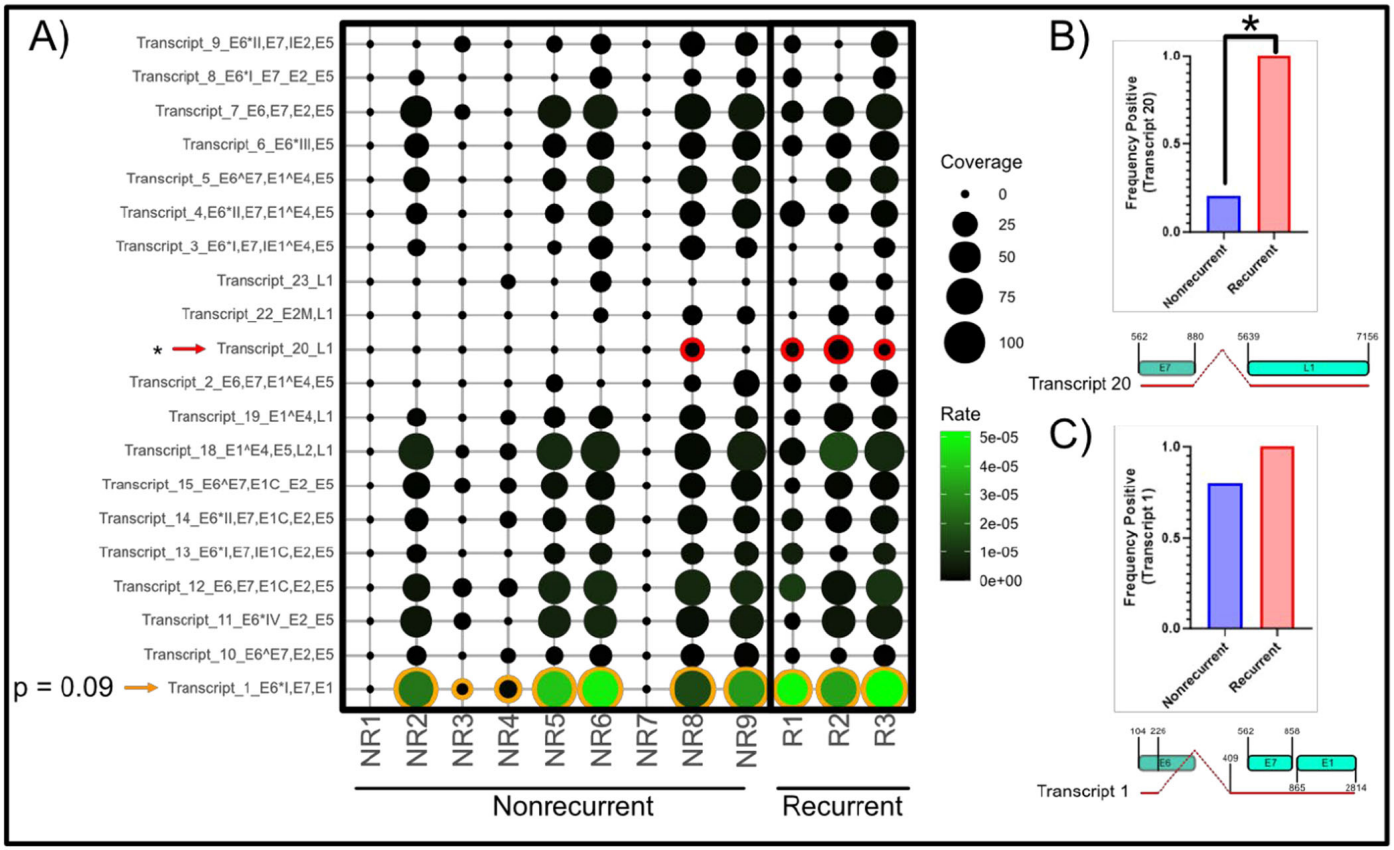
Relative abundance of HPV16 polycistronic transcripts in relation to anal cancer treatment outcome. Reads are aligned to polycistronic transcripts as opposed to a full ORF sequence. (A) Results in rate (v/h mapped read %) and coverage represented via bubble plot. Transcripts which significantly differ between groups are outlined in red (with red arrow) while those showing a trend are indicated with an orange circle and arrow (*p* < 0.1). Bar graphs indicating positive frequency in each group of the indicated transcript 20 (B) or transcript 1 (C) are shown. HPV16 sequence position numbers and transcript splice site as compared to HPV16 ORF sequence indicated for each transcript below the bar graph.

We next examined polycistronic spliced transcripts identified using an HPV16 transcriptome map reported in a prior publication [37]. We searched for sequences covering the spliced junctions to infer the prevalence of spliced transcripts in this cohort using a pipeline adapted from VIRTUS2 [36]. Transcript positions were initially adapted from the paper by Yu et al. [37], then reference sequences were constructed from these positional coordinates as spliced polycistronic full‐length transcripts. We detected the presence of most of the HPV16 spliced transcripts in at least one sample, though their magnitude (v/h reads) was low. Only the splice signature of transcript number 20 (nucleotides 562−858, 5639−7156) as identified in HPV16 transcription map was detected in all samples from the R group. Moreover, the transcription rate of transcript 20, encoding for “HPV16 E7^L1” open reading frames (ORFs), was significantly higher (by Mann−Whitney *U*‐test) in R group (3/3) but detected at only 1/9 in NR group (*p *< 0.05, Figure 3A,B). We also found that Transcript 1 (nucleotides 104−226, 409−2814), a spliced polycistronic transcript “HPV16 E6*I^E7^L1,” encoding, in order, for E6*, E7, and E1 was detected in both R and NR samples, with a trend for higher expression (*p*‐value 0.09) in the former (Figure 3C).

### 2.3 HPV16 (v/h Rate) Inversely Correlated With Host *HLA‐A* and Directly With DEAD Box Protein *DDX24* Expression in AC

**Figure 4:**
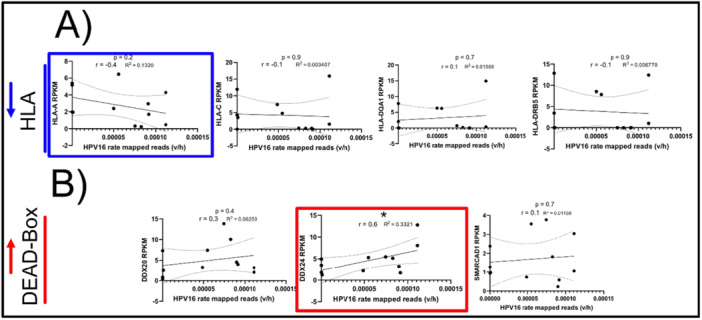
HPV 16 rate correlates negatively with HLA‐A and positively with DEAD‐Box protein (DDX24) gene expression. (A) Heatmap of HPV16/18 genome rate alongside expression levels of indicated HLA's or DEAD‐Box Protein family genes (indicated in RPKM, or rate of expression per kilobase exon per million reads). Mann−Whitney's *U* test (within Virtus2) or EdgeR differentially expressed gene *p* values are indicated between recurrent and nonrecurrent sample groups as indicated. Correlation analysis of HPV16 whole genome rate with indicated (A) HLA class I and II molecules or (B) DEAD‐Box proteins. R, R2, and *p* values for Pearson's correlation are indicated (*p < 0.05, **p < 0.01, ***p < 0.001, ****p < 0.0001 respectively).

Previously [31], we showed upregulation of key immune markers (encoding for HLA and dead box proteins) in the NR group. Because HPV16 was most ubiquitously identified in 83% of all samples analyzed, we wanted to determine whether HPV16 could also be interacting with the human transcriptome in a way that would be associated with non‐recurrence. We therefore examined whether the rate of HPV16 reads (using the whole HPV genome as reference, referred to as “HPV16 genome” later in this manuscript) correlates with the expression of these human transcripts. Among differentially expressed HLA transcripts, *HLA‐A* was found to inversely correlate with HPV16 rate, though this correlation was not significant (*p *= 0.2, *r* = −0.4) (Figure 4A). However, *DDX24*, a DEAD Box protein family subunit, directly and significantly correlated with the rate of HPV16 (*p *< 0.05, *r* = 0.6) (Figure 4B). These data suggest markers of HPV‐associated AC, irrespective of treatment outcome. This targeted analysis, although informative, was biased and did not provide the breadth of coverage to capture all genes directly or indirectly correlated with the HPV16 genome or transcript rate. Therefore, we next performed an unbiased bioinformatic approach to capture the correlation landscape of human genes whose expression increased with or decreased with HPV16 infection.

### 2.4 Unbiased High‐Throughput Analysis Determined That HPV16 and HPV16 Transcripts Correlates With Human Gene Ontology (GO) Signatures of Cell Growth

**Figure 5:**
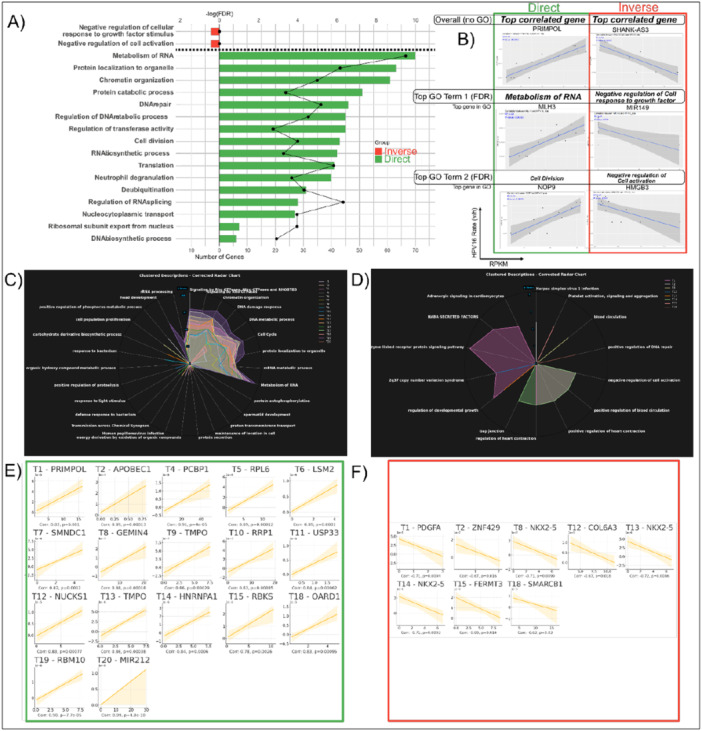
An unbiased HPV‐human gene correlation analysis indicates gene expression of HPV16 and its transcripts positively correlate with markers of cell growth in anal cancer. RPKM of human gene mRNA was correlated in an unbiased bioinformatic pipeline with the viral rate (viral/human reads) of all detected whole HPV type genomes as well as individual HPV16 transcripts. Then human genes which were directly or inversely correlated with HPV genome or transcript rates were enriched for gene ontology (GO) terms separately. (A) Pyramid plot of human genes which were inversely and directly correlated with HPV16 whole genome rate. # of genes enriching to GO term represented as bars, green for direct and red for indirect. (B) Correlation plot for highest overall directly correlated human gene with HPV16 rate (top row) as well as top gene correlated with HPV16 rate from top two GO terms (left, in green box). Lowest gene (top row), or highest inversely correlated gene with HPV16 rate as well as top inversely correlated gene with top two inversely correlated GO terms (right, in red box). Clustered radar chart of GO enrichment for all HPV transcripts (C) directly or (D) indirectly correlated human genes by transcript. # Genes in each GO term maps to radius of lines from center. Top (E) most directly or (F) most indirectly correlated gene for the highest gene # GO term of every transcript is represented as a faceted line plot with only line of best fit represented. Correlation and *p*‐value of each are also shown beneath each plot. “T#” refers to Transcript # of HPV16 individual transcripts identified in Figure 3.

To capture the full unbiased correlation landscape of all human genes which may potentially correlate directly or inversely with either (a) HPV16 rate (Figure 5A,B) or (b) individual polycistronic ORF‐encoding transcripts (Figure 5C−F), we developed a bioinformatic pipeline to determine the correlation between the rate of detection of HPV16 genome (full length HPV16 sequence used as reference) or individual HPV‐16 polycistronic transcripts (v/h rate) relative to expression (Reads Per Kilobase per Million mapped or RPKM) of human genes across all AC samples.

Examining at the HPV16 genome level, we found several genes, both studied (official HUGO Gene Nomenclature Committee [HGNC] symbol) and unstudied (only Ensembl ID such as ENSG00000259694, usually encoding for lncRNA or pseudogenes) that were differentially regulated. Interestingly, a higher number of genes showed a direct correlation (*N* = 1502) as compared to those showing an inverse correlation (*N* = 158) with HPV16 genome rate. Of the genes whose HGNC symbol was recognized and able to be enriched for GO signatures, directly correlated genes were found to be primarily those associated with cell growth with signatures of “Metabolism of RNA,” “DNA repair,” “Cell division,” Translation, and “Protein catabolic process” (Figure 5A) and this human transcript expression pattern is consistent with HPV infection induced upregulation of cell cycle and proliferation pathways in cancer.

Examining at the HPV16 transcript level, we found that the majority of human genes directly correlating with the various HPV16 transcripts also enriched to GO terms also associated with cell growth such as “Signaling by Rho GTPases,” “Metabolism of RNA,” “DNA metabolic process,” or “Cell cycle” (Figure 5C). The GO term with the highest number of directly (Figure 5C) or indirectly (Figure 5D) correlated human genes for the numbered HPV transcript shown (T1 is HPV16 Transcript 1, T2 is Transcript 2, and so on). The most directly (Figure 5E) or indirectly (Figure 5F) correlated human gene with each HPV16 transcript within the highest human gene number GO term are also shown as examples. Interestingly, not only is PRIMPOL the most directly correlated gene within the highest gene number GO term for HPV16 T1 (correlation = 0.82, *p* = 1.05E−03). It is also the most directly correlated human gene with both HPV16 T1 and HPV16 genome rate overall (correlation = 0.89, *p* = 1.17E−04). The most directly correlated human gene with HPV16 T20 which was found to be significantly enriched in R AC was also MIR212 as indicated (correlation = 0.99, *p* = 4.82E−10).

Lastly, although transcripts from the HIV‐1 genome itself were below the limit of detection, we investigated the “HIV‐1 Infection” GO term which was enriched from positively correlated genes for every HPV16 transcript and the HPV16 genome (full length HPV16 sequence used as reference) itself. Almost every HPV16 polycistronic transcript as well as the HPV16 genome itself had several genes which positively correlated with it enriching to this GO term. Those human genes enriching the term “HIV‐1 Infection” positively correlating with HPV16 whole genome transcription rates are shown in Supporting Information: Figure 3.

## 3 Discussion

In this study, using VIRTUS 2, we showed that HPV genotypes and their transcripts can be readily detected using bulk RNA sequencing data allowing for directly assessing, in the same cancer isolate, any associations between host and viral factors. The latter analyses show that it is possible to identify host factors whose expression is altered (upregulated/downregulated) by viral transcripts within the same AC isolate.

To date, it has been difficult to detect viral reads (which typically comprise less than 0.01% of total mRNA in any given human sample) in data obtained from bulk RNA sequencing. In recent years, several open‐source computational packages have been released allowing for examining the virome in data obtained from bulk RNA sequencing. The most common strategy has been to align reads from an entire bulk RNA‐sequencing sample to a given whole genome reference for a single virus, then count them. However, the problems with this approach were twofold. One is that many viral ORFs may have the potential to overlap with regions of the human transcriptome/genome and so disregarding this fact will result in overestimation of viral sequences. Second, most bulk RNA‐sequencing data sets are not sequenced to enough depth to accommodate the sensitivity required to detect viral transcript reads which are in comparison extremely low compared to any host transcripts. However, the tool we used, VIRTUS, alleviates the first issue by first aligning reads to the human genome, then taking any unmapped reads and aligning them to any viral reference it is given [36, 38]. The references used in VIRTUS2 contain more than 700 unique complete virus genome sequences including different HPV types to provide a complete picture of the “virome” in any given sample. Additionally, a novel feature of this package is the determination of a “rate” constant, which is used to normalize any mapped viral reads to the total number of mapped human reads in each sample (rate v/h or rate viral/human). Both strategies ensure that an accurate measure of viral reads relative to human reads is ascertained.

Detection of HPV16 as the predominant genotype in our study correlates well with what is reported in literature for other HPV+ squamous cellular carcinomas and determined by PCR based assays, thus indicating the feasibility of using bulk RNA sequencing for determining viral features [32, 39, 40, 41]. Furthermore, our approach is also sensitive to detect low frequency polycistronic transcripts such as HPV16 Transcript 20 encoding for E7^L1, which can perhaps be used as predictive biomarker of AC recurrence although our study is limited by small sample size and future studies will address whether this HPV transcript is unique to AC recurrence or other HPV associated human cancers observed in PWH and/or non‐PWH. Also, based on this study design, while these results seem predictive of recurrence, no isolates from the actual R cancer were procured. It will be important in future studies to compare and determine whether the HPV types observed and their transcript expression patterns between a primary and its R AC isolate are the same or differ.

It should be noted that in HPV+ tumors HPV DNA may exist in three forms: episomal, integrated into host chromatin, often near an actively transcribing gene, characterized with distinct virus−host junctions, or episomes of hybrid HPV‐host DNA or their combinations [42, 43, 44, 45]. In all HPV integration events, HPV‐DNA is truncated in E1 and/or E2 sequence and typically lack other downstream sequences such as L1 and L2 [40, 46, 47, 48]. In this context the detection of transcript 20 in AC is unexpected. Presumably, the transcript 20 (E7^L1) counts observed were indicative of new productive infection in some nontumor cells present in the same tissue along with the tumors. Transcript 1 (14–226^409–4237), the main source of E7 protein is typically most abundant in all high‐risk HPV infected cells (including HPV+ cancers). In contrast, the minor transcript 20 is considered to express L1 protein only in productive infection. From this data we infer that concurrent productive infection most likely occurred in AC isolates from patients who showed cancer recurrence, suggesting re‐infection perhaps reinvigorates a tumor promoting environment in these specimens. More extensive testing would however be required to confirm this hypothesis.

Our findings that HPV 16 showed a trend for an inverse correlation with HLA‐A expression suggests that HPV16 plays at least some role in the downregulation of HLA‐class I expression in AC to facilitate immune evasion from CD8 + T cell‐mediated cytotoxicity [49, 50]. This could also be due to alteration in the number and composition of immune cell populations in relation to HPV16 rates and location of tumor cells. However, confirmation of this interaction between HPV gene expression, localization of tumor, and immune cells would require spatial based single cell RNA‐sequencing analyses in multiple cancer samples. A direct correlation was also observed between a DEAD box RNA helicase, DDX24, and HPV16 genome rate in AC. DEAD box containing RNA helicases have both pro and antiviral roles [51]. Elevated DDX24 is significant for its role in negatively regulating RIG‐1 mediated innate immune response against cytosolic VSV RNA [52], while promoting DNA repair and cell cycle progression in vascular smooth muscle cells [53] as well as HIV replication [51, 54, 55]. Its expression was also found to be elevated in other cancers such as breast and gastric cancer [54, 56] where it was shown to control p53 activity and increased expression was associated with worse survival [54, 56]. Thus, further study on DDX24 expression is needed to unravel its potential role in HPV + AC. Additionally, though more HPV genotypes were identified in NR isolates, this observation was not statistically significant, most likely due to the small sample size. Also, of the observed non‐HPV16 genotypes, the majority that were found in most samples more than once were high‐risk types, mainly belonging to α‐9 group of E6/E7 phylogeny. The transcriptomic data on which this report is based is insufficient to determine status (integrated or secondary co‐infection) of these low frequency HPV types. Nevertheless, our study reveals possibility of detecting multiple HPV types from the bulk RNA sequencing data of archived formalin fixed AC specimens. In future studies, additional analysis could reveal correlation between altered expression of host genes, and transcripts of main (HPV16) and other coinfecting HPV types to determine their contribution to disease outcome.

Our unbiased correlation analysis between HPV and host transcripts showed that a majority of human genes positively correlated with HPV16 genome rate enriched to GO terms associated with cell growth, proliferation, or catabolic processes as expected for an oncogenic virus [57, 58]. While the HIV genome itself through VIRTUS2 was below the limit of detection for all samples (based on transcripts) within the isolates, 22 genes mapping to the “HIV Infection” term and therefore previously observed to be upregulated in active HIV infected samples were directly correlated with HPV16 genome rate, suggesting that perhaps HPV16, present in the cancer, is promoting an HIV‐permissive environment. For example, one of the genes identified in this study, BANF1, also known as BAF1, the host factor most positively correlated with HPV16 Genome rate in the “HIV Infection” GO term in this analysis, has previously been shown to be exploited by HIV to restore the activity of pre‐integration complexes (PIC's) and prevent their auto‐integration into themselves, thereby improving efficiency of integration into the host chromosome [59, 60, 61]. Identification of viral transcripts correlated with human genes regulating, cell growth, tumorigenesis, immune response, or HIV infection could inform antiviral and immunotherapeutic approaches as targets to reduce cancer recurrence. Additionally, host transcripts which are correlated with HPV16 genome rate may be necessary for HPV gene expression and functions of HPV proteins in AC or AINs. These host transcripts may be evaluated as potential therapeutic targets for HPV induced oncogenesis.

The current analysis has identified key host transcripts related to tumorigenesis (enriching to “cell growth”) correlating with HPV16 genome and transcript rates in all 12 AC isolates examined. Future studies would aim to evaluate the therapeutic potential of host factors, whose expression in R ACs is correlated with HPV16 transcripts rates to reduce incidence of recurrence.

One of our main study limitations is a small sample size for the R cancer group, and thus, future studies involving larger sample sets as well as longitudinal tumor samples from the same individual would be required to validate our study findings and to improve the generalizability of our findings at the population level. However, this does not affect the correlation analyses as these were not based on group comparisons. Another caveat is that with bulk RNA‐sequencing data, a direct assessment between host and viral factors cannot be performed at a single cell level. Nevertheless, single cell analyses‐based assays, although more informative, are expensive and thus bulk sequencing is a more economical choice due to its lower costs. Thus, the ability to examine both host and viral features in the same bulk RNA sequencing based data set will still yield more relevant information.

In summary, we have developed a unique approach to simultaneously analyze correlation between host and HPV gene transcription using the same bulk RNA sequencing data set from AC specimens. Future studies of spatial transcriptomics on FFPE cancer tissue will allow us to determine whether HLA‐A downregulation occurs in cells that are HPV infected and what impact this has on the proximity and function of tumor infiltrating lymphocytes. According to studies published on other SCC tumors using spatial transcriptomics, HPV diversity and heterogeneity exists between different cell type compartments and so this may be a more accurate representation of the AC landscape [62]. Follow‐up studies will involve a larger, well characterized cohort with longitudinal timepoints post initial CRT will confirm whether the candidate transcripts (host and viral) identified in this study are continually expressed and associated with recurrence of AC.

## 4 Methods

### 4.1 Cohorts and Study Design

The study cohort is described in detail in our prior work [31]. In brief, 12 AC patients (3 R and 9 NR) from the UAB O'Neal Comprehensive Cancer Center (UAB‐CCC) with electronic health records (EHR) from the University at Alabama Birmingham (UAB) HIV Clinic were reviewed by a licensed oncologist and confirmed by a pathologist. This study was approved by the UAB IRB Review Board. These archived FFPE primary tissues from individuals with confirmed AC diagnoses in the EHR were then requested from the UAB Tissue Biorepository and associated with the requisite metadata. For both R and NR, we used the primary tumor sample before the patient receiving CRT. Non‐recurrence is defined as the absence of disease at the site of the primary tumor and regional lymph nodes within 6 months from the end of CRT. Local recurrence (LR) is defined as persistent disease or recurrence at the site of the primary tumor. Each patient's EHR was retrospectively reviewed up to 5 years after the last session of CRT to determine if LR occurred.

### 4.2 RNA Extraction, Library Preparation, and RNA‐Sequencing

RNA was purified from FFPE tissues with the Quick‐DNA/RNA FFPE kit (R1009, Zymo Research). The concentration of RNA was assessed with a Nanodrop spectrophotometer. Libraries were prepared as previously described [31]. A 20 µL volume of at least 400 ng RNA was used for library creation. Libraries were generated with the SMARTer Stranded Total RNA‐Seq Kit v2–Pico Input Mammalian (Takara Bio USA). The pooled libraries were sequenced on a NovaSeq. 6000 (Illumina) to a depth of > 40 million paired‐end 150 bp reads for each sample. Phred quality score for all samples was good, with a mean of > Q30.

### 4.3 Bioinformatics Analysis

#### 4.3.1 VIRTUS2

Initial pre‐processing and analysis of FASTQ sequences for human mRNA gene expression was performed as previously described [31]. However, VIRTUS2 was used for whole genome splicing‐aware HPV type alignment and counting [36, 38]. The most recent human genome from the human genome project, GRCh38 was downloaded alongside the concatenated list of all HPV type genome sequences included within the VIRTUS2 repository. These viral sequences were acquired from the Virtect repository but originate from NCBI and PaVE. These references were first indexed with the createindex.cwl script and STAR to create an indexed human genome reference as well as “virome” genome reference containing whole genome sequences from > 700 viruses [63]. Donor library fastq files were trimmed to minimum sequence length of 75 bp and a Phred score cutoff of 30 using Trim Galore as previously described [31, 64]. A target file defines sequences as belonging to either R or NR groups. Singularity was used in place of Docker because of the lack of root privileges on the UAB Cheaha high performance computing (HPC) cluster. This was used alongside the Spliced Transcripts Alignment to a Reference (STAR) human and viral genome reference as well as the VIRTUS_wrapper.py python wrapper script to count viral genome reads as described in the VIRTUS2 repository. VIRTUS2 first filtered polyA/T sequences and mapped fastq sequences to human genome reference through STAR. All unmapped reads were then mapped to the indexed “virome” genome reference. Both coverage and rate viral/human (v/h) % reads were returned for all viral genome sequences contained in the reference fasta file. HPV type results above 0 rate v/h % and Coverage were then represented as a bubbleplot generated through R so as not to miss low frequency HPV types. “Rate v/h %” returns the viral reads for a given genome or transcript divided by the reads mapping to the human reference. For specific HPV16 ORF's or transcripts, additional fasta references were generated separately and then indexed with the same workflow. Virtus2 no longer supports gene‐level quantification and recommends using the original Virtus tool for this purpose. However, the original Virtus does not have a wrapper which performs analysis in a high‐throughput manner, so the same wrapper script used for genome quantification in VIRTUS2 was also used for ORF‐ and transcript‐level quantification, simply replacing the “virome” reference full of individual HPV type whole genome sequences with individual PaVE ORF gene or transcript sequences. Additional analyses and graphical representations were also performed using a combination of PRISM or R [65, 66]. HPV‐type specific ORF sequences obtained through the PaVE database for the HPV types previously identified as having detectable genome rates were used to create a targeted reference in place of the whole genome “virome” reference used previously. To ensure specificity and reproducibility, the whole genome sequences for these types were also included. All samples positive for an indicated HPV type ORF were positive for only the whole genome reference sequences of only those HPV types to which the ORF mapped (data not shown). The final analysis included all currently identified specific HPV16 polycistronic transcripts as described through PaVE [37].

#### 4.3.2 Correlation Analysis

Both human gene RPKM (HGNC symbols or Ensembl ID if HGNC symbol did not exist for a given reference transcript) and viral rate (v/h) for all HPV full genome or HPV16 individual transcripts were included in one Excel file. R was then used to compare every human gene RPKM value to every viral rate through a parallel processing script across all analysis pairs. For every pair, a corresponding correlation and *p* value of correlation was output, and those which were significant (*p* < 0.05) were also output into a separate sheet. GO analysis was performed through Metascape either as single analysis for direct or inversely correlated with HPV16 genome only, or using the “Multi‐Gene List” feature for all directly or inversely correlated transcript genes. Further generation of the Pyramid plot, radar charts, individual correlation plots, or panel correlation plots was performed through Python. Individual plots available on request.

## Author Contributions

Anju Bansal, Nilam Sanjib Banerjee, and Kevin J. Maroney conceptualized and designed the study. Kevin J. Maroney, Sadeep Shrestha, Nilam Sanjib Banerjee, Staci L. Sudenga, Sameer Al Diffalha, and Yuanfan Ye curated data and/or performed formal data analysis. Sadeep Shrestha, Kevin J. Maroney and Yuanfan Ye acquired funding. Anju Bansal, Sadeep Shrestha, and Nilam Sanjib Banerjee supervised the study and contributed to manuscript preparation. All authors reviewed and edited the manuscript.

## Conflicts of Interest

S.L.S. reports personal fees from Merck outside the submitted work. The other authors declare no conflicts of interest.

## Supporting information

Supplemental Figures Final.

## Data Availability

All raw fasta files, as well as processed data, are available through the Gene Expression Omnibus (GEO) repository (Accession number: GSE293618). Additional data including HPV type/transcript rates as well as correlation and significance values of the correlation analysis are available upon request.
