# Supplementary material for: Higher Expression of HPV16 Derived E7_LI Transcript Observed in Men With HIV and Recurrent Anal Cancer
Source: J Med Virol. 2025 May 3;97(5):e70371. doi: 10.1002/jmv.70371 (PMC12048892; doi:10.1002/jmv.70371)
Supplement: Supplementary file 1 — Supplemental Figures Final. [file JMV-97-e70371-s001.docx]

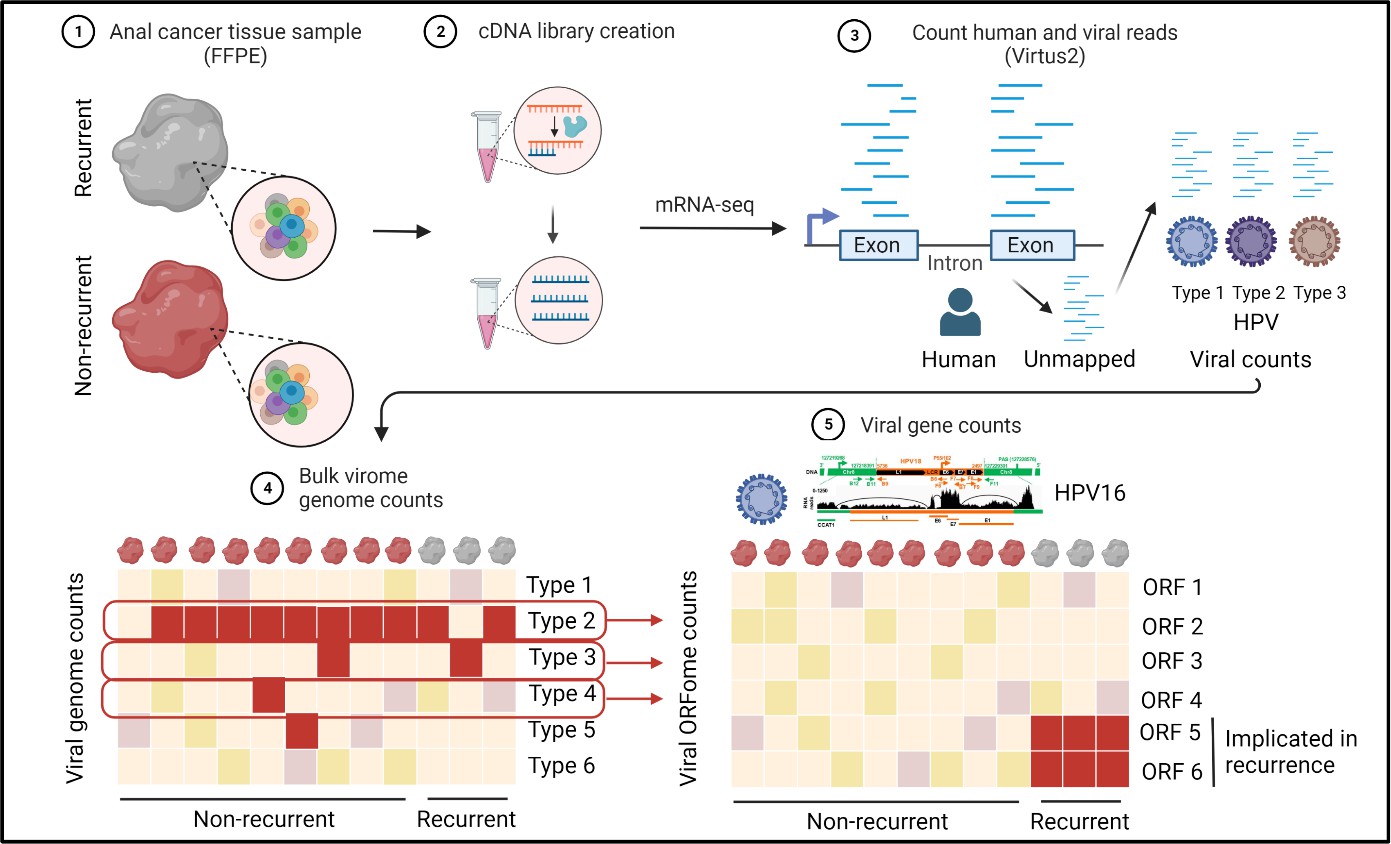


**Supplemental Figure 1: Graphical abstract of workflow used for viral detection**. Anal cancer FFPE isolates

**(1)** were first subjected to cDNA library creation in preparation for paired end bulk mRNA sequencing as done previously **(2)**. The Virtus2 wrapper for parallelized group-defined paired-end analysis was used to first map reads in these samples to the most updated publicly available human reference genome before unmapped reads were then mapped to all HPV types identified so far through Virtus2 internal reference genome **(3)**. Most represented HPV types were then assessed for impact on SCCA and/or recurrence status (**4)**. Reads were then again mapped through a modified of the same pipeline, but this time to identified transcript sequences of each HPV type from the PaVe database and again assessed for impact on SCCA and/or recurrence **(5)**.


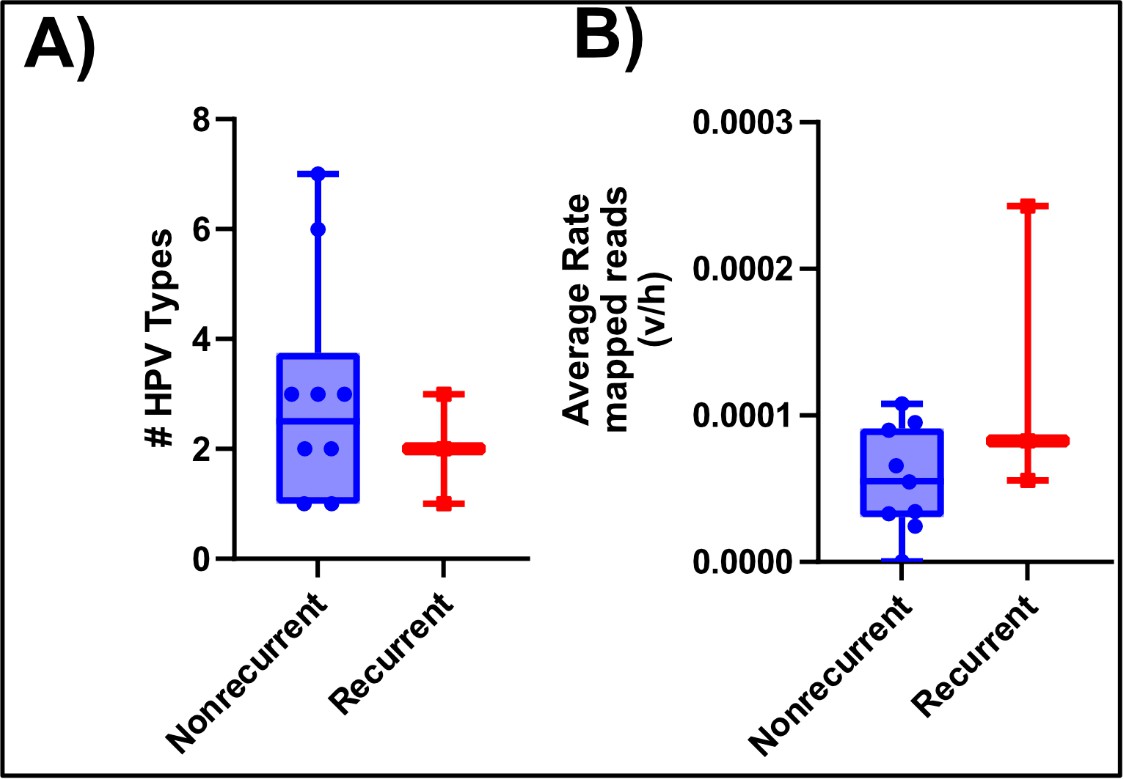


**Supplemental Figure 2: Number of HPV types and average rate mapped reads are similar between groups.** Box-plot of **A)** # HPV types for whose whole genome reads rate was detected in each group. **B)** Average rate mapped reads for all whole genome HPV rate reads detected by sample in each group.


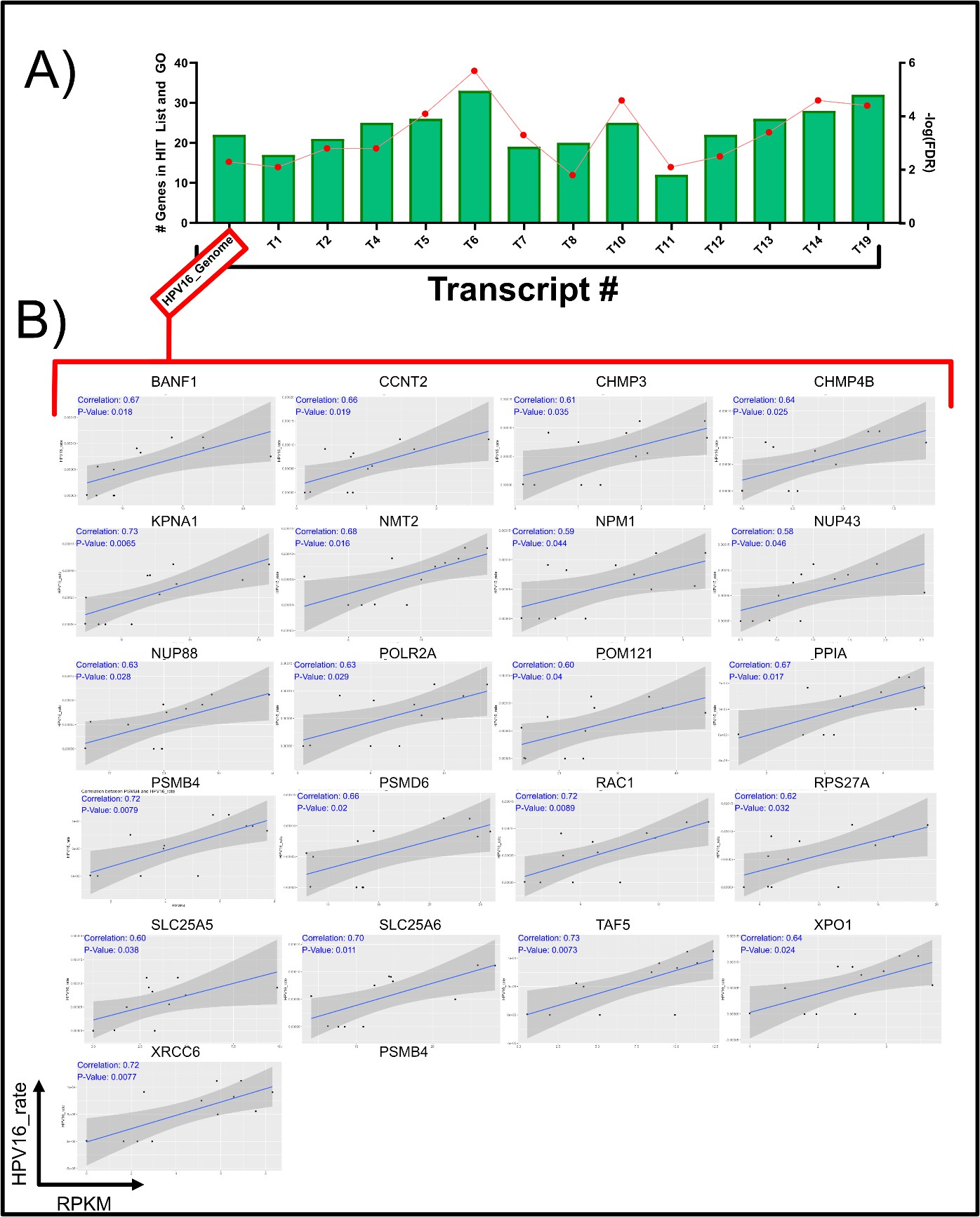


**Supplemental Figure 3: Human genes associated with HIV infection which positively correlated with HPV16 genome rate. A)** The number of genes associated with the GO term “HIV Infection” in positively HPV16 genome or HPV16 transcript (T1-T19) correlated genes as well as the -log(FDR) value for the GO term by genome or transcript is shown as a bar graph for # genes overlaid with a line graph for -log(FDR). **B)** All positive correlation graphs with correlation and p value are indicated by gene for all genes enriching to the GO term “HIV Infection” in the HPV16 genome condition.
